# The application of SWAT+ model to quantify the impacts of sensitive LULC changes on water balance in Guder catchment, Oromia, Ethiopia

**DOI:** 10.1016/j.heliyon.2022.e12569

**Published:** 2022-12-22

**Authors:** Bekan Chelkeba Tumsa, Goshu Kenea, Bayisa Tola

**Affiliations:** Faculty of Civil and Environmental Engineering, Jimma University, Oromia, Ethiopia

**Keywords:** Guder catchment, LULC changes, SWAT+, Water balance components |

## Abstract

Global population growth and scarce resources increase the competition for land use. Despite the fact that the impacts of climate change have been recognized, the conversion of LULC is still often neglected and threatens catchment hydrology. This is mostly seen in the developing world, where agriculture is the crucial source of their food security. The conversion of LULC has been jeopardizing water balance components and damaging ecosystem. This study demonstrates the application of SWAT+ in quantifying the impacts of LULC changes on the Guder catchment water balance. The impacts were quantified between 2003 and 2021, and the watershed experienced an increase in agriculture and settlement while forest, shrubland, and wetlands declined. The time-series-based performance of the SWAT + model shows the model is more restructured and capable of simulating streamflow compared to observed during calibration and validation. In this long-term evaluation, the model simulates changes in runoff of 56.5%, water yield of 65.2%, lateral flow of 21.6%, percolation of 46.2%, return flow of 76.4%, and ET of 0.2% between 2003 and 2013. Moreover, some attributes of the water balance have increased from 2013 to 2021, with runoff of 34.3%, water yield of 2.3%, ET of 4.5%, and lateral flow of 72.6%. However, as a result of increasing settlement, which reduces infiltration through interceptions and converts rainfall to runoff, percolation and return flow were decreased by 45.6% and 86.7%, respectively. Water yield and runoff show a linear relationship with changes in LULC, and the most sensitive land use changes that affect them are agriculture, forest, and settlements. The simulation results show a water balance deficit under the impacts of LULC changes in the third simulation. Furthermore, the increased surface of runoff has been limiting the amount of groundwater recharge into the soil and reducing return flow and percolation in the second simulation.

## 1 Introduction

Climate change is emerging and becoming more widespread around the world in the twenty-first century as a result of naturally induced factors and anthropogenic activities [1]. The change in climate variability has been affecting the hydrological components and water balances of many watersheds worldwide [2]. This in turn brought about the shortage of water demand for irrigation, water supply, hydropower, and unexpected flood hazards [3]. On the other hand, land use and land cover change are affecting biodiversity, ecosystem function, and the life of fauna as a result of agricultural and settlement expansion [4]. A change in LULC affects rainfall patterns, which cause the alteration of water balance by affecting the critical hydrological components such as surface run-off, groundwater recharge, infiltration, interception, and evapotranspiration [5, 6].

Indeed, the socio-economic movements of the population are major factors that cause rapid LULC dynamics [7]. The developing world, especially the African continent, is one of the victims of both climate and LULC changes as their food security has been dependent on agricultural business [8]. The change in LULC has received less attention, but it threatens water balance and major catchment hydrology by lowering infiltration rates, evapotranspiration, and soil water storages [9]. Despite the fact that climate change is a threat, LULC change is still playing a significant role in climate acceleration [10]. Because the variability of LULC will affect streams, groundwater recharge, rivers, and other water bodies [11]. However, the sensitivity and response of each LULC to changes in hydrological components and cycles are not similar [12]. This shows the response of land use changes to water balance and other water resource components depends on the characteristics of hydrological response units (HRUs) [13]. Indeed, the patterns of the hydrological cycle depend mainly on the intensity of rainfall, the magnitudes of evapotranspiration, and the behavior of LULC, plus the progress of climate change [14].

On the other hand, global water balance significantly depends on the intensity, duration, and characteristics of hydrological cycles [15]. Once the hydrological cycles are affected due to either natural processes or human-induced factors, the capacity of the water balance to sustain life remains in question [16]. Hence, the investigation of concurrent LULC changes and the impacts of each variability on each hydrological component will improve the estimation of potential consequences both in space and duration [17]. As a result, water resource planners and managers must quantify the potential impacts of LULC changes on water balance and the hydrological cycle [18]. According to many sources of literature, the potential impacts of LULC change on water balances have recently been understood and have become a hot topic to be addressed [19]. Afforestation, agricultural land expansion, and settlement are critically increasing in the developing world, as reported in [20]. The restructured version of the SWAT model (SWAT+) is known for its effectiveness and efficiency for the simulation of complex hydrological processes under the impact of LULC changes [21, 22]. As a result, the primary goal of this research is to use the SWAT + model to predict and quantify the effects of each LULC change on Guder catchment water balances in the upper Blue Nile River basin.

## 2 Description of study area

### 2.1 Location

**Figure 1:**
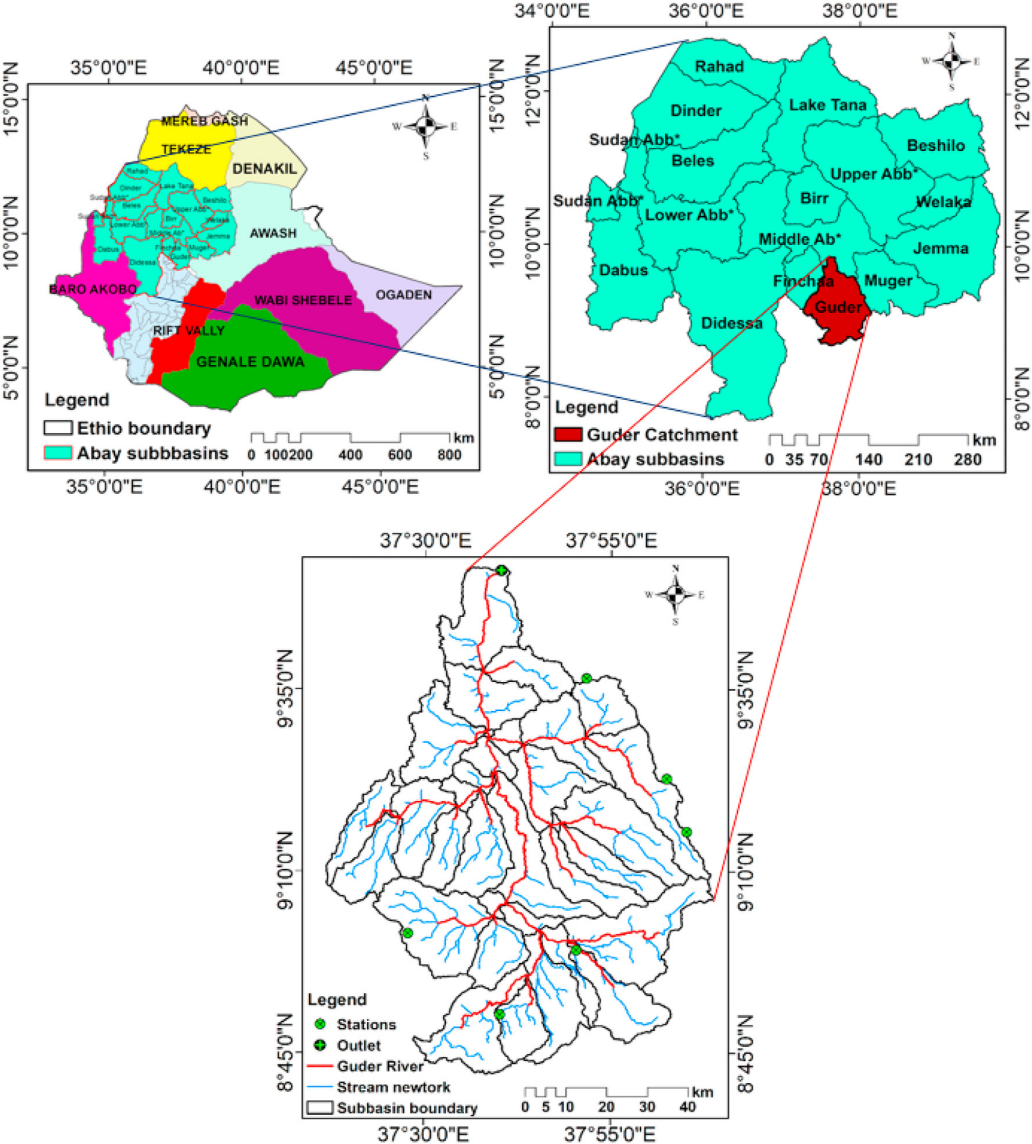
Map of area under study.

Guder catchment is located in the upper Blue Nile River basin in Oromia regional state, with latitudes ranging from 7^0^30^0^ to 9^0^30^0^ N and longitudes ranging from 37^0^00^0^ to 39^0^00^0^ E, as shown in Figure 1. The total area of the catchment is estimated at 6764.7 km^2^. The middle and upper parts of the catchment are known for their high agricultural and pastoral activities. Due to the reliance of the population settled in this catchment on agriculture, a massive land use dynamic was investigated [23].

### 2.2 Weather conditions

Seasonal rainfall characterizes the catchment in tropical wet to semi-arid and arid climate regions. Due to its high topographical setting that ranged from 850 m to 3500 m, the catchment has a mild climate, with the highest rainfall occurring from May to September, an annual average value of 1228 mm, and average maximum and minimum temperatures of 21.47 °C and 9.82 °C, respectively. The dry season in the catchment lasts a long time, from November to April, with moderate rainfall in the middle of February and April.

### 2.3 Sources of data's

This study uses three types of data, namely, spatial data (classified land use and land cover, soil map, and DEM), meteorological, and hydrological data. The major SWAT + inputs related to meteorological data on a daily basis are precipitation, max and min temperature, sunshine hours, relative humidity, and wind speed from six synoptic stations, except for temperatures from five stations situated in the catchment for the years 1992–2020. For the years 2000–2020, daily Streamflow data collected at outlet stations was used for SWAT + calibration and validation in SWAT + Toolbox v0.4.5. All recorded data and a raster soil map were collected from the Ethiopian Minister of Water, Irrigation, and Electricity (MoWIE). The topographic data (digital elevation model) with 12.5 m resolution was downloaded from the Alaska website (https://asf.alaska.edu/) to develop topographic features such as floodplains and terrain settings.

### 2.4 Soil map

**Figure 2:**
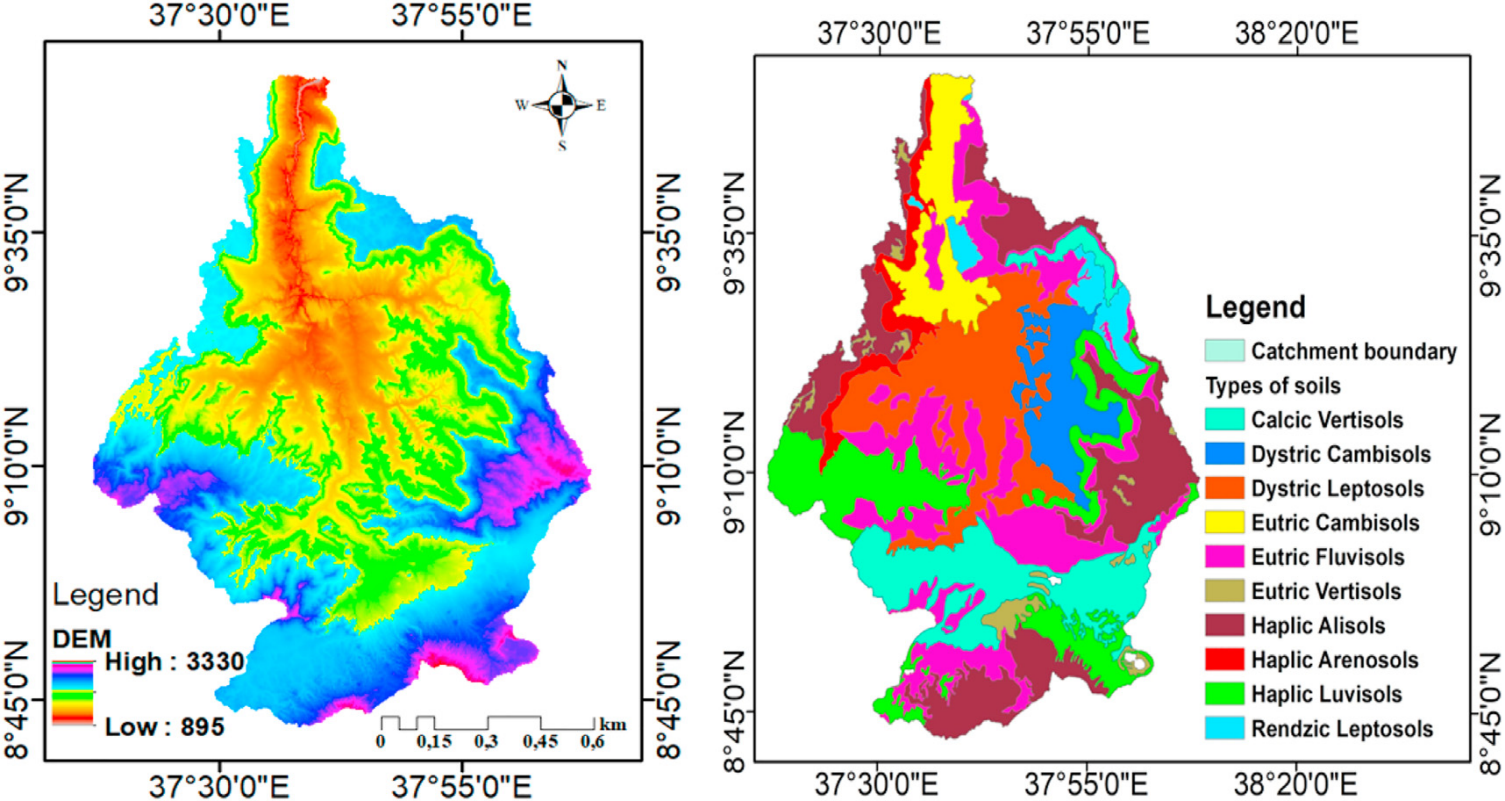
Maps of the major soil classes and DEM of the study area.

The Guder catchment has been covered by thirteen soil types distributed at each HRU. This class of soils has its own effects on water balances and hydrological components. However, each soil category found in the catchment's hydrological units (HRUs) has a distinguished response to hydrologic variables. The dominant soils in the Guder catchment are Haplic Alisols and Eutric Fluvisols, with total area coverage of 18% and 20%, respectively (Figure 2). The least common soil types that covered the minimum area were Eutric Vertisols, Haplic Arenosols, and Rendzic Leptosols, each with 3% as shown in Table 1.

**Table 1:** Major soil type distributed in the study area.

No	Major soil types	WRB_Group	Soil code	Area (km^2^)	% Coverage
1	Calcic Vertisols	Vertisols	VkVr	865	13
2	Dystric Cambisols	Cambisols	RdCm	408	6
3	Dystric Leptosols	Leptosols	RdLp	983	15
4	Eutric Cambisols	Cambisols	VeCm	427	6
5	Eutric Fluvisols	Fluvisols	ReVr	1297	20
6	Eutric Vertisols	Vertisols	VeVr	119	2
7	Haplic Alisols	Alisols	VhAl	1213	18
8	Haplic Arenosols	Arenosols	RhAr	224	3
9	Haplic Luvisols	Luvisols	RhLv	968	14
10	Rendzic Leptosols	Leptosols	RkLp	194	3

## 3 Methodology

### 3.1 LULC classification and accuracy assessment

**Figure 3:**
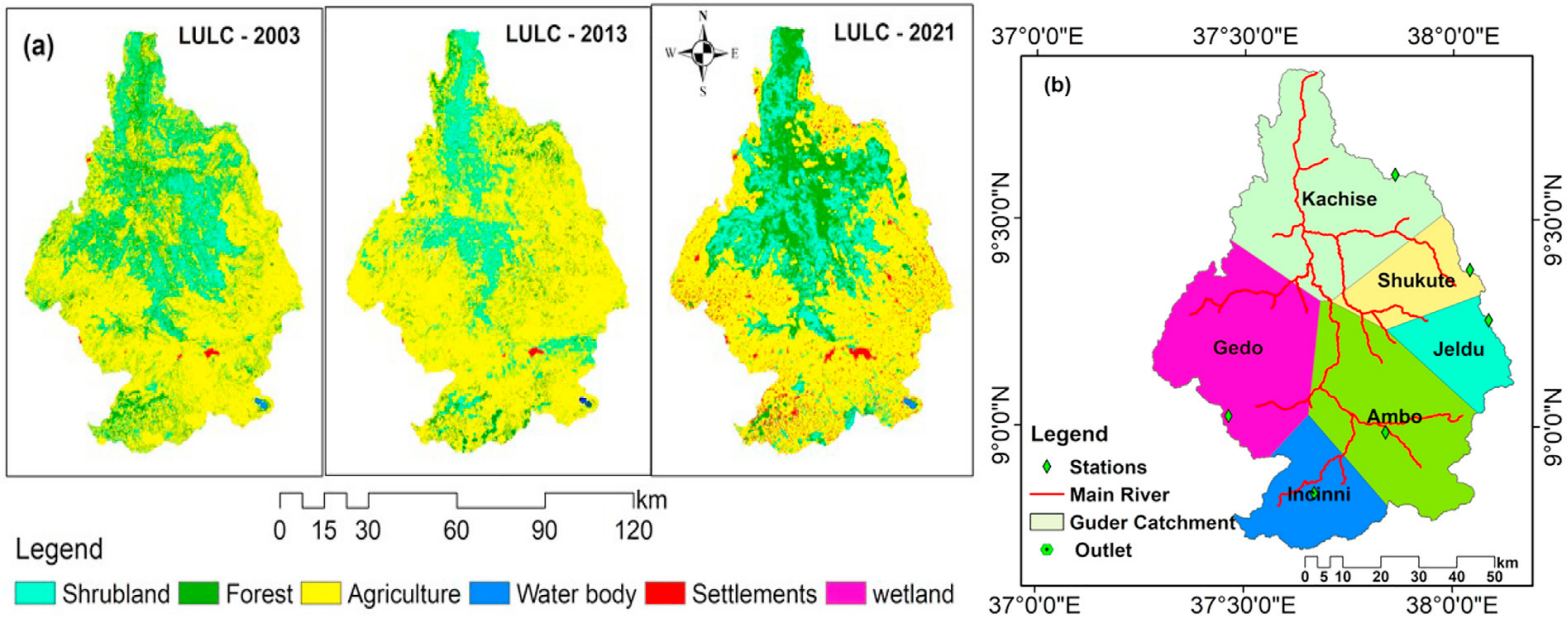
Types of Land use and land cover (a) and Thiessen polygon for annual rainfall of stations (b).

The satellite image of LULC data may be distorted and need to be processed prior to use as an input for hydrological modelling [22]. This will make the study more reliable and accurately represent the actual impacts of a LULC scenario on hydrological processes and water balances. The satellite image of LULC was downloaded from the USGS website with selected sensors, namely ETM+, TM, and OLI, as shown in Table 4. The procedures of pixel cell mosaic and layer stacking were done using ERDAS 2015 software for further image classification and accuracy assessment. In this study, 1635 ground truth points were taken for each image to increase its accuracy relative to its ground truth, as seen in Table 2. This assessment is to realize how well the pixels were sampled into the actual land use and land cover types, as seen in Figure 3(a). User accuracy, producer accuracy, and Kappa coefficient statistics (K) were used to assess the accuracy of the final image classification as shown in Table 3 and computed using Equations (1) and (2).

(1) $$k=\frac{N\sum_{\;i=1}^{\;r}X_{ii}-\sum_{\;i=1}^{\;r}\left(X_{i+}*X_{1+}\right)}{N^{2}-\sum_{\;i=1}^{\;r}(X_{i+}*X_{i+})}$$

(2) $$Overall\;accuracy=\frac{Number\;of\;points\;correctly\;classified}{Total\;number\;of\;points\;classified}$$

where N is the total number of sites in the matrix, r is the number of rows in the matrix, $X_{ii}$ is the number in row i and column i, $X_{i+1}$ is the total for row i, and $X_{i+}$ is the total for column.

**Table 2:** Confusion Matrix for Land use and land cover of 2021.

Class Name	AGRL	FRST	RNGB	WATL	WETL	SETL	Total
AGRL	**365**	10	5	0	0	0	390
FRST	10	**107**	16	1	0	3	147
RNGB	10	0	**350**	0	6	18	410
WATL	0	0	14	**108**	21	0	143
WETL	0	12	3	21	**82**	0	152
SETL	0	1	18	1	0	**393**	413
Total	395	131	406	131	109	416	**1635**

**Table 3:** Summary of LULC classification accuracy assessment.

LULC Types	2003				2013				2021			
	PA%	UA%	OA%	KC%	PA%	UA%	OA%	KC%	PA%	UA%	OA%	KC%
Agriculture	0.91	0.91	0.90	0.89	0.91	0.91	0.91	0.9	0.94	0.94	0.92	0.9
Forest	0.82	0.73			0.9	0.84			0.82	0.73		
Shrubland	0.84	0.85			0.78	0.78			0.86	0.86		
Water body	0.9	0.85			0.81	0.74			0.82	0.76		
Wetland	0.79	0.79			0.87	0.72			0.75	0.84		
Settlement	0.87	0.88			0.9	0.91			0.92	0.89		

Note: UA user's accuracy, PA producer's accuracy, OA overall accuracy, KC kappa coefficient.

**Table 4:** Satellite imagery data for LULC.

Scenario	Bands	Sensor types	Path/Row	Acquisition date	Resolution	Cloud cover (%)
2003	7	ETM+	172/055	22/12/2003	30m	<1
2013	8	TM	158/064	31/12/2013	15m	<1
2021	8	OLI	169/059	01/12/2022	15m	<1

The rate of change of land use and land cover for this study area was calculated using the following Equation **(3)** for different land uses.

(3) $$r=\left(\frac{1}{y_{2}-y_{1}}\right)x\;\ln\left(\frac{A_{2}}{A_{1}}\right)$$

Where, *r* is the change for each class per year, $A_{2}$ and $A_{1}$ are the classes of areas at the end and the beginnings of years, respectively for the period being evaluated, and *t* is the number of years spanning that period.

### 3.2 Estimation of areal rainfall to test each station's contribution

**Figure 4:**
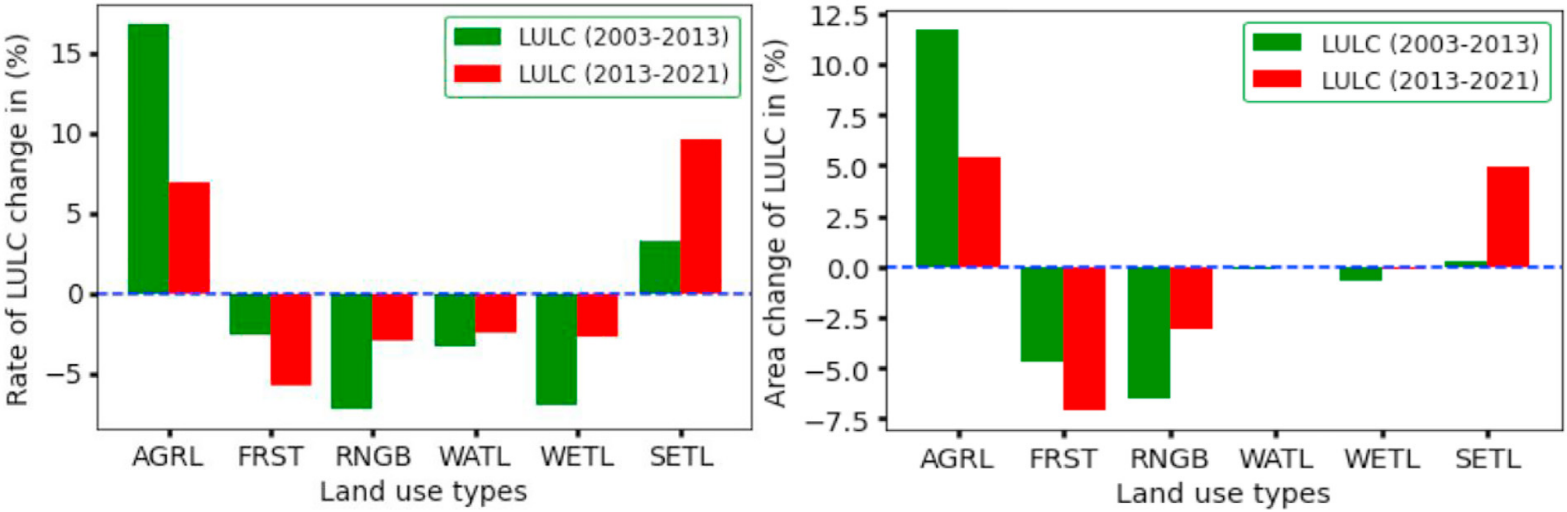
Area and rate of change of LULC in the catchment.

The gauging stations for rainfall in the catchment only provide a point sample of precipitation. This recorded rainfall value should be translated to an average value to estimate the contribution of each available station, as shown in Figure 4(b). Hence, the Theissen polygon is the well-known method that evaluates the contribution of each station to the catchment in developing weight average factors by assuming the rainfall is the same at any place nearest to the gauging point [24]. In general, this method used equation (3) to estimate the mean aerial rainfall close to the gauge. In general, this method used equation **(3)** to estimate the mean aerial rainfall close to the gauge.

(4) $$P_{av}=\frac{\sum_{i=1}^{n}P_{i}A_{i}}{\sum_{i=1}^{n}A_{i}}$$

where $P_{av}$ represent average areal rainfall (mm), $P_{i}$ is precipitation of stations 1, 2... n, respectively and $A_{i}$ area coverage of stations 1, 2, 3…, n in the Theissen polygon. The method gives weight to each station's data in proportion to the space between them. Kachise, Ambo, and Gedo stations are among those with the largest area coverage of the watershed, with 26.29%, 23.23%, and 23.12%, respectively, as seen in Table 5.

**Table 5:** Aerial coverage and annual rainfall contribution of the stations.

Polygon	Stations name	Latitude (^o^)	Longitude (^o^)	Area (km^2^)	Weighted average (%)
W	Ambo	8.99	37.84	1571.41	23.23
2	Gedo	9.02	37.46	1563.75	23.12
3	T/Incinni	8.84	37.67	737.78	10.91
4	Jeldu	9.26	38.09	517.83	7.66
5	Kachise	9.61	37.86	1778.37	26.29
6	Shukute	9.78	38.04	594.58	8.79

### 3.3 Description of SWAT + model

The SWAT + model is a QGIS extension that integrates a wide range of available geospatial data to represent the characteristics of the watershed at the hydrological response unit (HRU) level rather than the sub-basin level [25]. In the model, the impacts of spatial heterogeneity of topography, land use, soil, and slope on catchment hydrology were described in subdivisions at the watershed level [26]. The Guder catchment was divided into 33 sub-basins, each with its own set of 184 landscape units and channels. SWAT+ is a restructured version of SWAT (Soil Water Assessment Tool). The model is very effective in assessing the impacts of climate change and LULC on surface and subsurface hydrological processes [27]. It estimates and simulates hydrological components at the hydrological response unit (HRU) level, which include a variety of land uses, soil types, and slopes [28]. Mostly, land use and routing units are involved to simulate the hydrological processes in SWAT+ [29]. In SWAT+, the hydrological cycle and processes were simulated using the water balance Eq. (5). Furthermore, water yield is one of the most important components that determine the availability and sustainability of water resources in the catchments. It is computed from equation (6), which aggregates surface runoff, lateral flow, Tloss and return flow.

(5) $$W_{yld}=Q_{surf}+Q_{lat}+Q_{gw}-T_{loss}$$

(6) $${SW}_{t}={SW}_{0}+\sum_{i=1}^{t}\left(R_{day}-Q_{sur}-E_{a}-W_{seep}-Q_{gw}\right)$$

Where $S_{Wt}$- soil water content (mm), $S_{Wo}$- soil water content on day i (mm), t-time (days), $R_{day}$ - precipitation on day i (mm), $Q_{surf}$- surface runoff on day i (mm), $E_{a}$- evapotranspiration on day i (mm), $W_{Seep}$ - water entering the vadose zone from the soil profile on day i (mm), and $Q_{gw}$- return flow on day i (mm), $T_{loss\;}$ is the transmission losses (mm) and $Q_{lat}$-lateral flow (mm).

### 3.4 Model calibration, validation, and sensitivity analysis

There are various sources of uncertainty that are related to data and model assumptions. The sensitive parameters have an immediate impact on streamflow and water balance. In order to overcome the model uncertainty, these sensitive parameters should be identified, calibrated, and validated for the entire hydrological component that operates in the catchment. Those parameters that affect calibration were adjusted through a trial-and-error procedure until the acceptable range between estimated and observed discharge was reached. In SWAT+, the well-known sensitivity analysis methods are Sobol, Fourier amplitude, Random Balance Design Fourier amplitude, and Delta moment independent measures [30]. Because of its popularity in estimating significant sensitive parameters based on the P-factor and t-test, the Sobol method was chosen. The SWAT + Toolbox version v0.4.5, which is an independent tool from SWAT+, was used to calibrate and validate streamflow [31]. The performance of the model was evaluated using a statistical measure indicator, as shown in Eqs. (7, 8, and 9).

(7) $$R^{2}=\frac{\sum_{i=1}^{n}\left(O_{i-}O_{ave}\right)\;x\;\left(S_{i}-S_{ave}\right)}{{\left(\sum_{i=1}^{n}{\left(O_{i}-O_{ave}\right)}^{2}\right)}^{0.5}\;x\;{\left(\sum_{i=1}^{n}{\left(S_{i}-S_{ave}\right)}^{2}\right)}^{0.5}}$$

(8) $$BIAS=\frac{\sum_{i=1}^{n}S_{i}-\sum_{i=1}^{n}O_{i\;}}{\sum_{i=1}^{n}O_{i}}\;x\;100\;\%$$

(9) $$NSE=1-\frac{\sum_{i=1}^{n}{\left(O_{i}-S_{i}\right)}^{2}}{\sum_{i=1}^{n}{\left(O_{i}-\dot{O_{i}}\right)}^{2}}$$

${Where\;O}_{i}\;and\;S_{i}\;are\;observed\;and\;simulated\;values,\dot{O_{i}}\;is\;mean\;values\;of\;observed\;data,n-is\;the\;total\;numbers\;of\;data$

### 3.5 Limitation of the study

The SWAT + model is used to analyze the hydrological processes and phenomena at HRU and land scape units (LSU) levels, which are composed of varied objects such as LULCs, soil types, and slopes that are more complex to analyze and interpret the results. However, despite its complexity and unsuitability for interpretation, the output is more reliable at HRU than in the subbasin. This is one drawback of the new version of the SWAT model.

## 4 Result and discussions

### 4.1 LULC changes over two decades

The Guder catchment has experienced land use and land cover changes since 1996, when agricultural land expansion and settlement were advancing. The rate of change of agricultural land from 2003 to 2013 was 16.8% and 6.94 % from 2013 to 2021. This indicates that the catchment has been under high pressure from socio-economic mobilizations as the food security of the population heavily depends on agriculture activities. Much forestland and shrubland have been converted to agricultural land. Forest land has decreased by 4.74% between 2003 and 2013, and 7.12% between 2013 and 2021. This show huge movement of people to the catchment site and deforestation activities has been increasing. This intensive land use change has been exposed the watershed to high surface runoff and soil erosion, which brought about sediment loading into the entire watershed. In this study, the evaluation was based on three classified LULC of different years by considering DEM, soil map and climate data remain constant and land use land cover was changing. According to the classified image of the LULC from 2003, agricultural land accounted for approximately 64.1% of the total area, forest land accounted for 21%, and settlement accounted for less than 1% of the total area of the catchment. After nearly two decades, agricultural land accounts for 81.2% of total land, forest land accounts for 9.15%, and settlement accounts for 5.94% of total land. The other land use and land cover classes show a decreasing trend in the last two decades, as shown in Table 6 and Figure 4.

**Table 6:** Annual rate of land use change.

LULC Class	2003	2013	2021	Annual Change (%)		Rate of Change (%)	
	Area (km^2^)	Area (km^2^)	Area (km^2^)	2003–2013	2013–2021	2003–2013	2013–2021
AGRL	4333	5127.3	5496	11.74	5.45	16.80	6.94
FRST	1421	1100.5	619	-4.74	-7.12	-2.56	-5.75
SHRB	852	410.0	203	-6.53	-3.06	-7.30	-3.02
WATL	25	18.0	14	-0.10	-0.06	-3.29	-2.51
WETL	87	43.2	33	-0.65	-0.15	-7.00	-2.69
SETL	49	68.0	402	0.28	4.94	3.28	9.54

### 4.2 Model calibration, validations and sensitive analysis

The SWAT + model has the capability to calibrate streamflow and prioritize the parameters that affect flow, water balances, and major hydrological components. The simulated water balance components such as surface runoff, lateral flow, percolation rate, return flow, precipitations, and evapotranspiration are adjusted when the observed flow is calibrated with the simulated flow to test the model's performance. This indicates that sensitive parameters have the potential to affect hydrological processes and need to be prioritized with their fitted values. The calibrated SWAT + model with the recorded streamflow at the outlet of the Guder catchment prioritized twelve parameters as the most sensitive parameters that affect hydrological simulations. The calibrated streamflow and simulated flow show that the data closely matched the observed flow over the entire period. The performance of the model was evaluated using statistical indices, which showed good agreement between them with ${Pearson\;correlation\;coefficient\;of\;\left(r\right)=0.86,R}^{2}=0.74,NSE=0.76,BIAS=-12.37)\text{.}$

**Figure 5:**
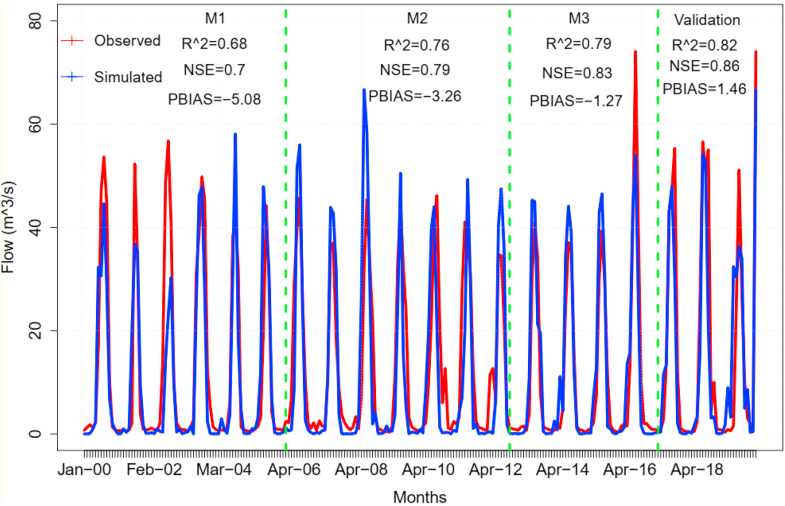
Calibrated and validated Streamflow under the impacts of LULC changes.

The validation of the model indicated that the simulated and recorded data were more in agreement with $(r=0.94,R^{2}=0.82,NSE=0.86,BIAS=-7.46$ as shown in Table 8 and Figure 5. However, the calibration and validation did not show a consistent trend, with overestimates at some events and underestimates at others for the entire calibration process. The calibration process was done by calibrating the water balance and streamflow for daily conditions through trial and error by changing the SWAT + parameters' values within their acceptable ranges to optimize the model. The calibration and validation process has been performed to predict and adjust annual water balance components such as surface runoff, water yield, evapotranspiration, percolation rate, lateral flow, return flow, and daily precipitation.

The optimized parameters that fit the calibration process were the SCS runoff curve number for moisture condition II (cn2), the soil water factor for curve number III (cn3_swf), the scope of the baseflow alpha factor (alpha_bf), the groundwater contribution to streamflow (mm H2O) (gwflow_lte), the depth of water in the shallow aquifer required for return flow to occur (gwflow_lte), Minimum water depth in the shallow aquifer required to return flow (mm H2O) (flo_min), universal soil loss equation p-factor (usle_p), and minimum depth of water in the shallow aquifer for percolation to the deep aquifer to occur (mm H2O) (revap_min). Groundwater "revap" coefficient (revap_co), surface runoff lag coefficients (surlags), plant uptake compensation factor (**epco**), and soil evaporation compensation factor (esco) are the most sensitive parameters that affect water balance based on their maximum and minimum values set by SWAT + output as depicted in Table 7.

**Table 7:** Sensitive parameters and their calibrated value.

number	Parameters	object	Min-value	Calibrated value	Max-value	Units
1	cn2	hru	35	46	95	
2	cn3_swf	hru	0	0.287	1	
3	alpha	aqu	0	0.52	1	Days
4	bf_max	aqu	0.1	1.05	2	Mm
5	gwflow_lte	hlt	0	7.34	10	mm-H2O
6	flo_min	aqu	0	0.03	0.5	M
7	usle_p	hru	0	0.72	1	
8	revap_min	aqu	0	26.9	50	mm H2O
9	revap_co	aqu	0.02	0.18	0.2	
10	surlags	bsn	0.05	13.4	24	Days
11	epco	hru	0	0.8	1	
12	esco	hru	0	0.675	1	
13	perco	hru	0	0.24	1	fractions

Note: **hru**-hydrological response unit, **aqu**-aquifer, **hlt -hru_lte)**, **bsn**-basin.

**Table 8:** Performance of the SWAT+ during calibration and validation with statistical parameters.

Years of Calibration	$R^{2}$	NSE	BIAS	r
2000–2006	0.68	0.7	-5.08	0.86
2007–2012	0.76	0.79	-3.26	0.9
2015–2020	0.79	0.83	1.27	0.93
Validation (2012–2015)	0.82	0.86	-7.46	0.94

### 4.3 Simulated water balance components

The water balance in the catchment is composed of different major hydrological components. Each of these water balances has been affected by the land use and land cover dynamics in the Guder watershed. The simulated scenario for each LULC change in a sub-watershed was simulated and calibrated by the SWAT + model, which proves the major components of water balance were affected. The resulting simulation based on three different LULC (2003, 2013, and 2021) revealed that almost all water balances and major hydrological components were changed. The average annual value of precipitation in the catchment shows a decreasing trend of 2.06% (1439.39mm–1410mm) in 2003–2013 and 1.55% (1410mm–1388.36mm) in 2013–2021. This decreasing trend of precipitation in the watershed has a clear implication that the changes in LULC are affecting the hydrological cycle and hydrological processes for the sustainability of water balance.

**Figure 6:**
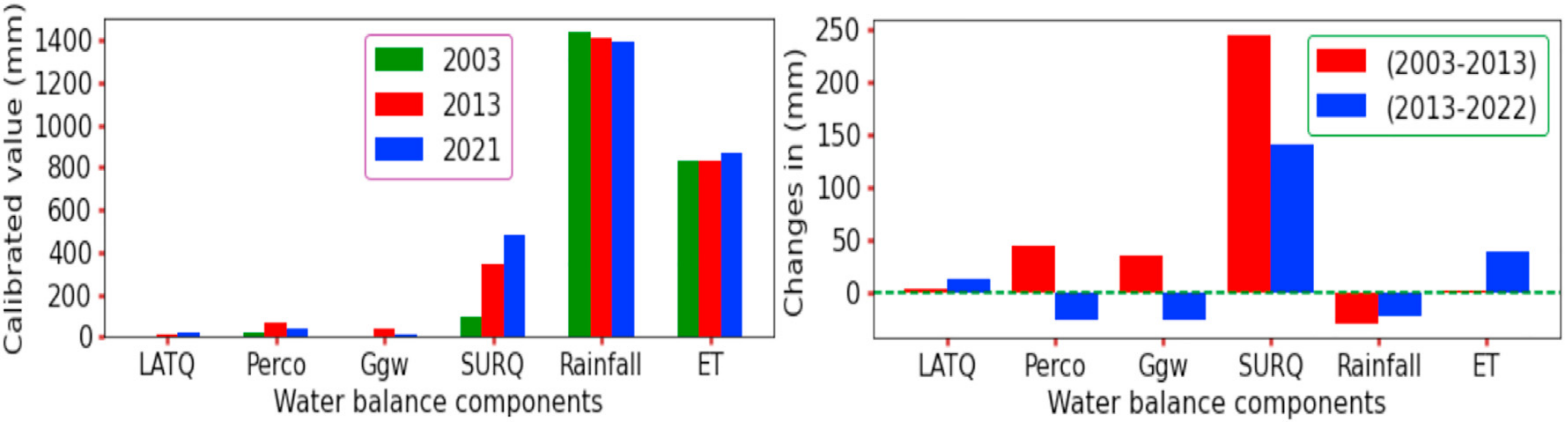
Water balance components under LULC change from (2003–2021).

Surface runoff, on the other hand, has increased significantly from 2003 to 2013, by 244.56 mm (56.52%), and by 140.39 mm (34.3%) from 2013 to 2021. The reason for the increase in surface runoff was due to the expansion of agricultural land and deforestation in the watershed. However, the rate of percolation increased by 46.15% (43.52mm) in the first decade and decreased dramatically by 45.5% (25.54mm) in the second decade. Furthermore, the return flow has increased by 76.39% in the first decade and decreased by 86.7% in the second decade. This implies that the groundwater percolation capacity into the soil has been limited by the change in LULC within the catchment and has caused streamflow and channel loss. Indeed, an increase in water yield in the Guder catchment of 65.21% from 2003 to 2013 and 2.3% from 2013 to 2021 has implications for simulated groundwater recharge, which was mandated to be reduced in the second decade. In this study, the mean annual change of surface runoff and water yield was more pronounced than changes in other hydrological processes. The simulated hydrological process for each year was quantified to determine the contribution of all components to the overall annual average water balance, as shown in Figure 6 and Table 9.

**Table 9:** Water balance components and their change under LULC variability.

WBC (mm)	Years			Changes (mm)		Changes in (%)	
	2003	2013	2021	(2003–2013)	(2013–2021)	(2003–2013)	(2031–2021)
Rainfall	1439	1410	1388	(-) 29.4	(-) 21.6	(+) 2.1	(+) 1.6
SURQ	94.1	338.6	479.0	(+) 244.6	(+) 140.4	(+) 56.5	(+) 34.3
LATQ	6.9	10.6	22.7	(+) 3.8	(+) 12.1	(+) 21.6	(+) 72.6
PERCO	25.4	68.9	43.4	(+) 43.5	(-) 25.5	(+) 46.2	(-) 45.5
ET	829.2	831.1	869.2	(+) 1.9	(+) 38.1	(+) 0.2	(+) 4.5
$Q_{gw}$	5.5	41.3	16.3	(+) 35.8	(-) 25.0	(+) 76.4	(-) 86.7
Water yield	106.5	390.5	518	(+)284	(+)127.5	(+)65.21	(+)2.3

**WBC**-Water balance components, **SUR_Q**-surface runoff, **WYLD**-Water yield, **LAT_Q**-lateral flow, **PERCO**-Percolation, **Q_gw**- Return flow, **ET**-evapotranspiration.

Since the objective of this study is primarily to quantify the impacts of LULC change on water balance components, the simulation has revealed that some hydrological components are increasing with LULC changes. Hence, as LULC changes from 2003 to 2021, there is expected to be either an increase or decrease in the style of runoff and curve numbers (CN). For this catchment, agricultural land, deforestation, and settlement were rapidly increasing. Therefore, the areas of 4333 km^2^ of agriculture in the first case and 5496 km^2^ in the third case will not generate the same magnitude of runoff, or CN. The same is true for settlement expansion in the catchments, where an increase in impervious area increases the likelihood of runoff and CN. This increase in runoff and other components is strongly related to the sensitive LULC change that triggered and initiated runoff and other hydrological processes.

### 4.4 The relationship of water balance components with LULC changes

In nature, land use and land cover change have their own sensitivity to affecting water balance components. The magnitude of each LULC change will determine the catchment water budget and other major hydrological processes. This reveals that each land use and land cover type might have a different relative sensitivity to runoff generation and other hydrologic processes. However, the response of water balance components to LULC change also depends on climate conditions, soil types, and slope of the sub-watershed, as concluded by [6]. In this study area, agricultural land and settlement area were increased, and it's the most pronounced LULC change between 2003 and 2021. In this case, the expansion of agricultural land facilitates the generation of surface runoff, water yield, and soil erosion, which in turn increases sediment load. On the other hand, the increasing settlement in the catchment leads the watershed to develop more rainfall interceptions areas, which affect infiltration, groundwater recharge, lateral flow, percolation, and return flow. However, considering that the magnitude of changes in each water balance cannot exceed the calibrated value in a decade, the annual exceedance of each water balance component with respect to changes in LULC in 2003, 2013, and 2021 was more visible and reliable.

**Figure 7:**
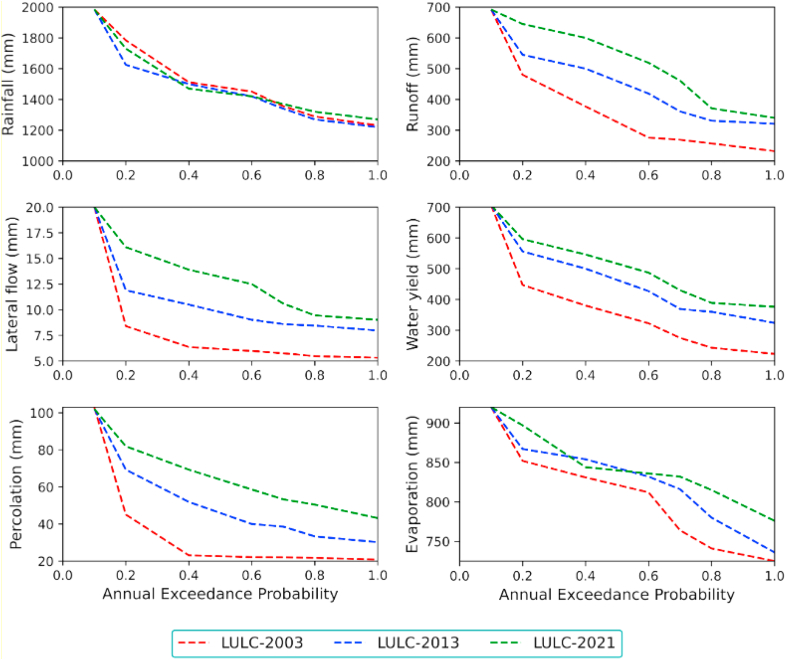
Annual exceedance probability of water balance components under LULC change.

In this analysis, the annual exceedance probability of each water balance exceeding its annual maximum value was below 20% for all components except rainfall and evapotranspiration. This impact was anticipated as a result of the marginalized effects of both climate change and land use variability. However, the exceedance probability of other water balance components exceeding their maximum value in the years 2003–2013 and 2013–2021 was more than 50%. Moreover, surface runoff and water yield in this catchment were increasing along with the change in LULC scenarios. The annual exceedance probability reveals the significant relationship between LULC change and water balance components, as shown in Figure 7. As a result, different land uses and land covers have responded to water balance components and major hydrological processes in different ways. This study summarized that best land use management in watershed regions, with effective implementation, can reduce the influence of land use change on water balance components.

### 4.5 The impacts of each LULC changes on water balance and water yield

In the last 18 years, the Guder catchment has experienced large land use changes. Each year's classified satellite image shows a high rate of conversion from one land use to another. However, land uses have different behaviors and capacities in response to hydrological processes. Because of the increased settlement caused by massive population movement and intensity in the catchment, agricultural land was increased. Due to this land use conversion, surface runoff increased by 221.8 mm from 2003 to 2013 and by 14.7 mm between 2013 and 2021 in the area covered by agricultural lands. With the same area, water yield increased by 261.3mm and 1.8mm in 2003–2013 and 2013–2021, respectively.

**Figure 8:**
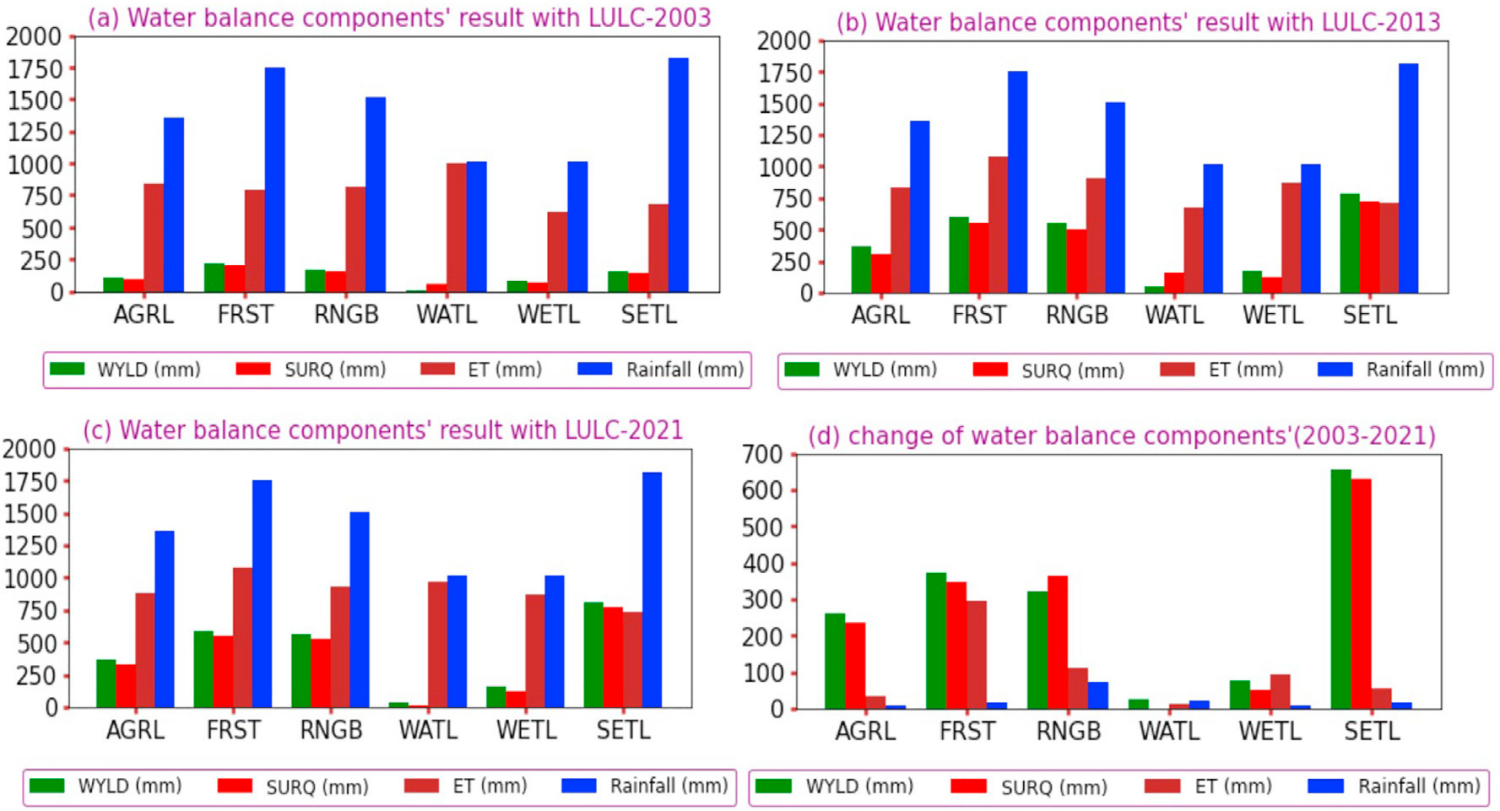
The impacts and sensitivity of each LULC on water balance components.

On the other hand, deforestation has contributed to a massive change in runoff and water yield. Surface runoff was increased by 346.15 mm between 2003 and 2013 and water yield was decreased by 12.9 mm from 2013 to 2021 due to deforestation for agricultural expansion. Moreover, the rise of settlements causes high surface runoff and water yield from the catchment, with 587mm and 626.6mm between 2003 and 2013, respectively. Evapotranspiration and rainfall have been rarely affected by land use and land cover changes and have shown decreasing trends in the last two decades. Furthermore, agricultural land, forests, shrubland, and settlements were the most sensitive LULC changes to water balance components. Water yield, surface runoff, evapotranspiration, return flow, and groundwater recharge (aquifer) were the most identified water balance components affected by land use and land cover changes in the Guder watershed, as shown in Figure 8.

### 4.6 Spatial map of water balance components simulated with the LULC of 2003

The impacts of land use in a sub-watershed on the water balance component vary with respect to the distribution of HRU's. The influences of each LULC change are determined by the characteristics of each HRU in the sub-watershed region. In this case, 64.03%, 21%, 12.6%, and 1.29% of the catchment were covered by agriculture, forest, shrubland, and wetland, respectively. Under the influence of each HRU, which was made up of different LULC, soil, and slopes, these hydrological components were spatially distributed and varied in the catchment.

**Figure 9:**
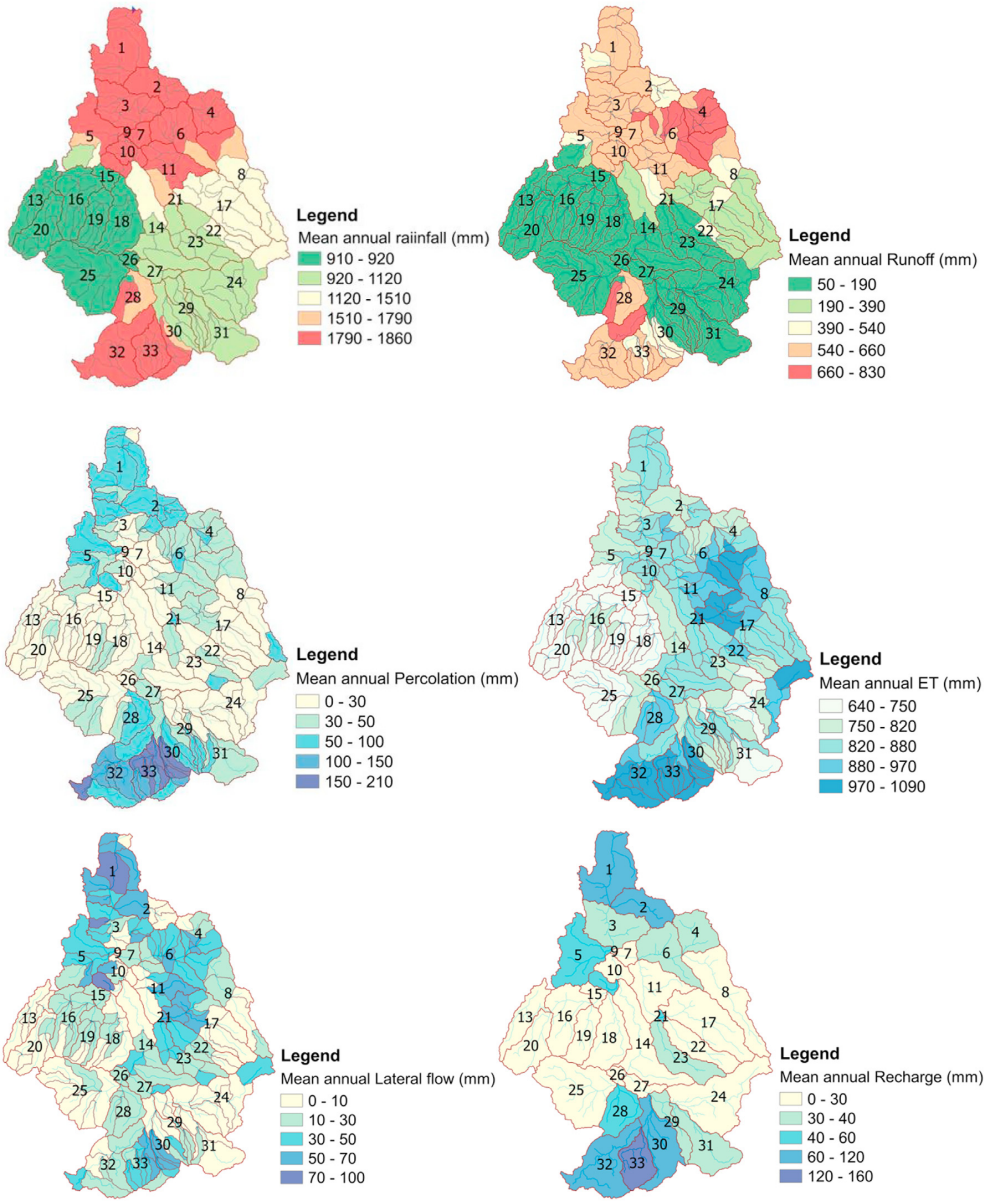
Spatial maps of water balance components under the influence of LULC 2003.

**Figure 10:**
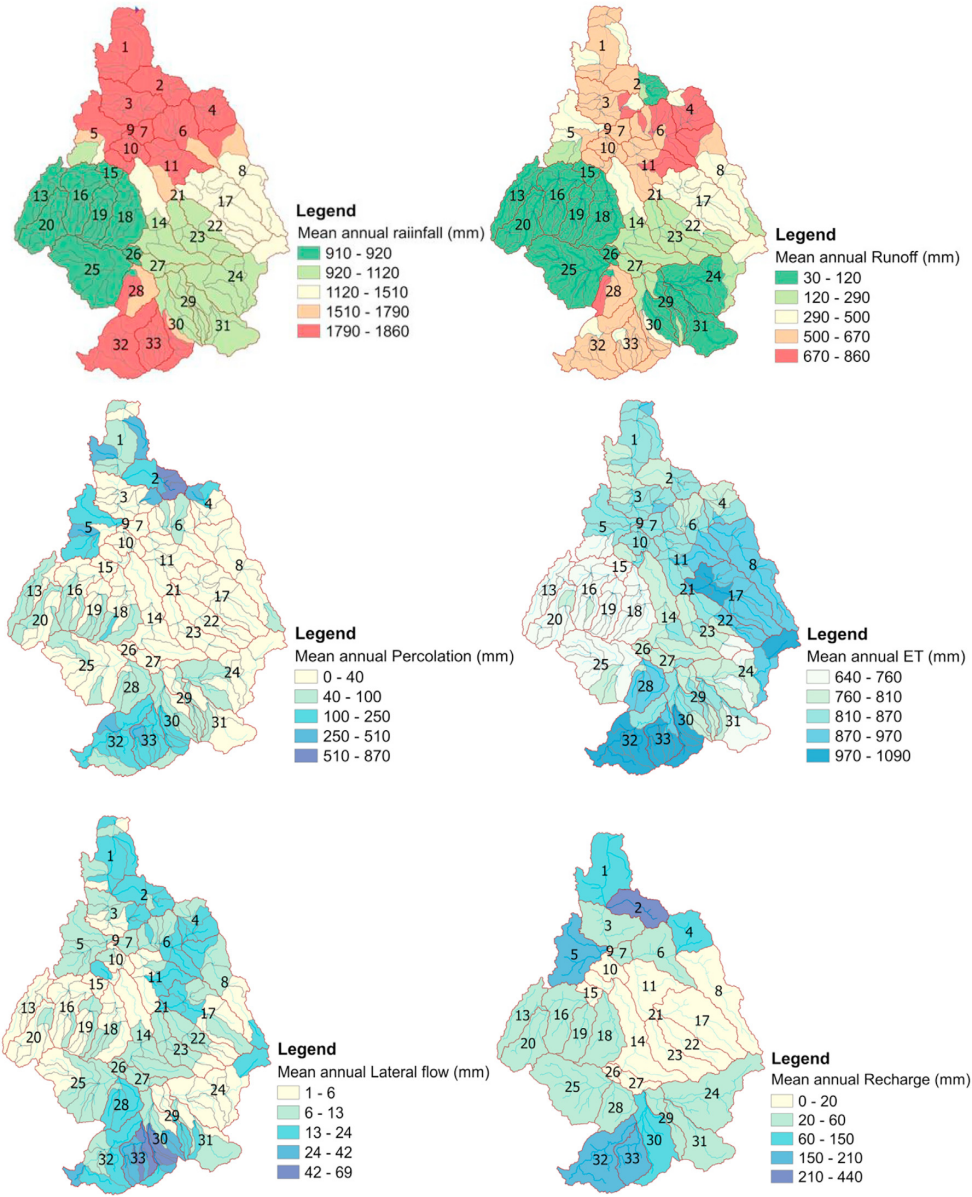
Spatial maps of water balance components under the impacts of LULC 2013.

In this simulation, the water budget ratios were 0.25 (streamflow to rainfall), 0.02 (percolation rate to rainfall), 0.8 (ET to rainfall), 0.88 (runoff to total flow), and 0.12 (base flow to total flow). This indicates that the water balance was safe (+478.4 mm) even though rapid hydrological response changes have taken place with considerable LULC changes. However, the streamflow budget was reduced by 25.9% due to total streamflow loss, 8.83% due to evaporation, and 17.11% due to seepage. As shown in Figure 9, the spatial distribution of the water balance component has followed the footprint of rainfall distribution. The maximum mean annual rainfall was seen at the upper and downstream parts of the catchment, and other water balances’ maximum mean annual value was also concentrated in the same direction as rainfall. This shows a strong relationship between precipitation and hydrological processes. Furthermore, the upper and downstream parts of sub-basins were the sensitive regions and prone areas to surface runoff, with shrubland and agriculture covering much of the land use. The simulated maximum mean annual evapotranspiration was recorded in the northern and eastern parts of the catchment (see Fig. 10).

### 4.7 Spatial maps of water balance components under the impacts of LULC 2013

The reflection of LULC sensitivity to catchment hydrology depends on the intensity of its influence on hydrological processes. In each hydrological response unit of the Guder catchment, the impacts of LULC were greater than those of soil and slope. Because the rapid expansion of agricultural land and settlements played a great role in varying water balance and other hydrological processes, in this scenario, 75.8%, 16.3%, 6.06%, and 1% of the total area were covered by agriculture, forest, shrubland, and settlements, respectively. Agricultural land and settlement show a similar increasing trend as of the first simulation done with LULC in 2003. This caused an increase in all water balance components by more than 52 percent except rainfall and evapotranspiration, as shown in Fig.**10**. In this case, the water budget ratios were 0.28 for streamflow to rainfall, 0.05 for percolation rate to rainfall, 0.6 for ET to rainfall, 0.87 for runoff to total flow, and 0.13 for base flow to total flow. This ratio indicates the relationship among water balance components and that runoff and total flow are highly correlated. In this case, the water balance is again safe (+119.4 mm), even though sensitive hydrological processes were affected by the LULC changes.

Furthermore, the streamflow budget was diminished with total streamflow losses of 10.59%, evaporation losses of 3.87%, and seepage losses of 6.93%. The behaviors of the spatial rainfall distribution were similar to the first case with LULC in 2003. However, surface runoff increased from upstream to downstream through the middle parts of the catchment. All sub-basins reside in the upper, lower, and middle parts of the catchment, which are exposed to high flooding and considered to be flood-prone areas. The spatial maps indicate an increase in surface runoff, percolation, and return flow. However, the map indicates lateral flow was decreased at sub-basin level and evapotranspiration was shifted from one sub-basin to the other with a slow rate of increment. This is due to the conversion of large areas of shrubland and forest to agriculture and settlements.

### 4.8 Spatial map of water balance components under the impacts of LULC 2021

The catchment was dominated by agricultural land with 81.3%, shrubland with 3%, and forest area with 9.15% in the current LULC conditions. The components of the water balance, such as surface runoff, lateral flow, evapotranspiration, and water yield, still showed an increasing trend. Rainfall intensity, percolation, and return flow were decreasing. Indeed, the cause for decreasing rainfall is not only accelerated by land cover variability. However, climate change, which requires separate investigation, plays a significant role in influencing the hydrological cycle, which in turn influences rainfall.

**Figure 11:**
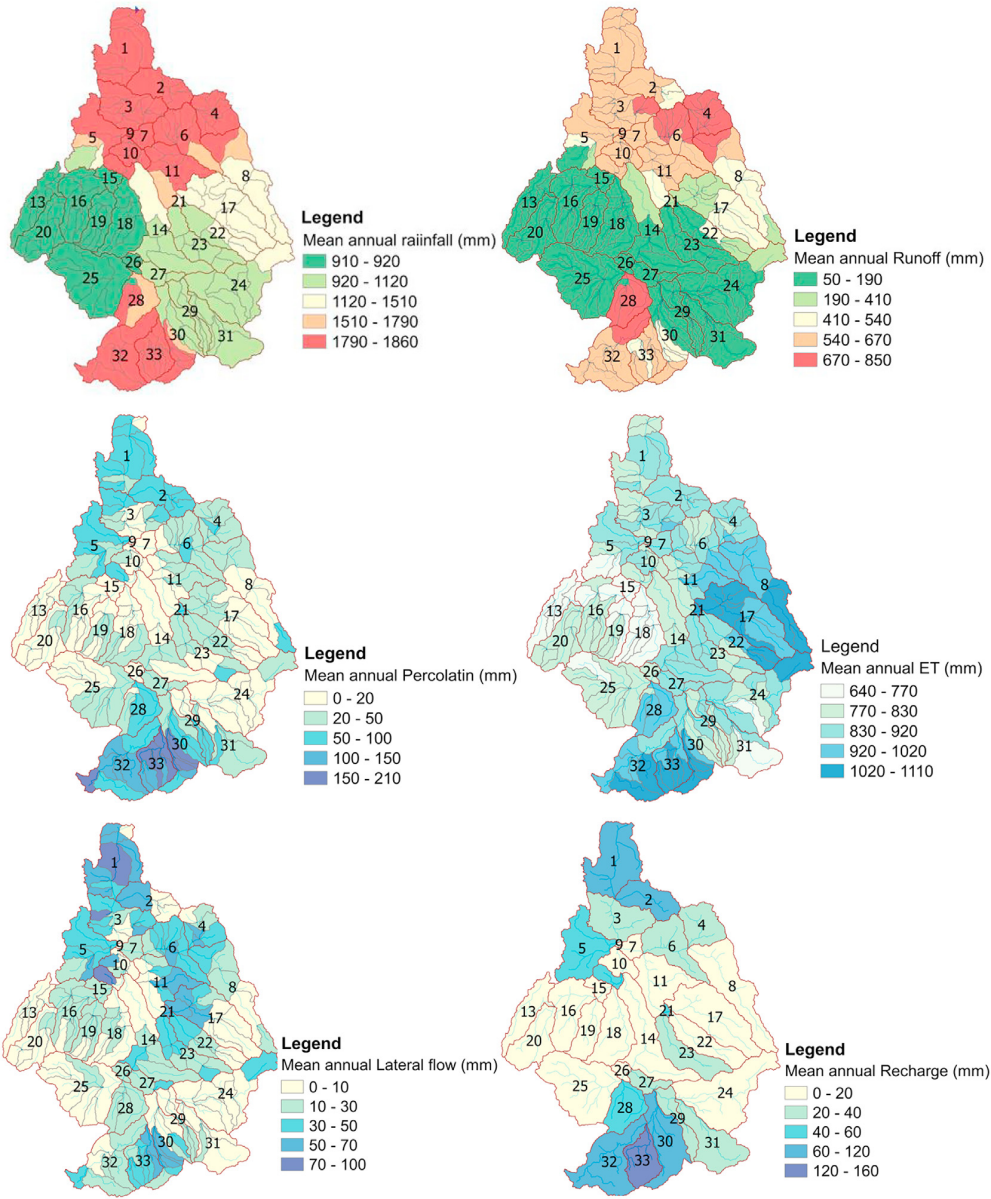
The distribution of water balance components under the impact of LULC 2021.

In this simulation, the impacts of the LULC change were more visible, as surface runoff and water yield were slowly increasing with 140.4 mm and 11.3 mm, respectively, relative to the previous simulated result with the LULC of 2013. This condition developed as the catchment recovered from deforestation and agricultural expansion in the middle and lower reaches. However, water balance was not maintained and was depleted by 42.3 mm, which needs immediate measurement with land use management. Moreover, the streamflow budget in the catchment was diminished with streamflow loss, evaporation loss, and seepage loss of 11.72%, 4.79%, and 6.9%, respectively. The spatial maps of all water balance component distributions in Figure 11 show more about the effects of historical LULC changes in the Guder sub-watershed.

In this simulation, the water budget ratios were 0.28 for streamflow to rainfall, 0.03 for percolation rate to rainfall, 0.63 for ET to rainfall, 0.90 for runoff to total flow, and 0.1 for base flow to total flow. This ratio indicates the relationship among water balance components, runoff, total flow, and ET to rainfall is highly correlated. This ratio shows the linear relationship among water balance components. The strong relationship of surface runoff with rainfall intensity in all simulation periods shows that the catchment might be exposed to high flooding events in the near future.

## 5 Conclusions

So far, the response of sensitive LULC change to water balance components and streamflow has been investigated with climate data from 1992 to 2020. in case large LULC dynamics took place in the Guder catchment between 2003 and 2021 and caused the diminishing of streamflow, evaporation, and seepage loss. The contribution of each LULC in affecting water balance components was identified, and the level of impact has been summarized through spatial maps. The large changes in land use that impacted the water balance component were concluded with the massive expansion of agriculture and settlement areas, which generate surface runoff, water yield, and soil erosion.

On the other hand, the removal of large areas of forest and shrubland causes an increase in surface runoff and water yield in the lower and upper parts of the catchments. This critically threatens lateral flow, groundwater recharge, return flow, and percolation rate. Surface runoff and water yield increased with medium range in the first LULC simulation in 2003. However, the spatial maps in the second simulation show that all water balance components were increased, particularly surface runoff by 56.5%, water yield by 65.2%, and return flow by 76.4%. In both simulations, the mean annual water balance was positive despite some deficits observed in a few sub-basins at downstream parts. A water balance deficit was observed in the third simulation with LULC of 2021. The cause of this deficit was highly related to the increasing runoff and the decreasing return flow, lateral flow, and percolation by 86.7%, 45.5%, and 45.6%, respectively. This result indicates that the current water balance situation is in jeopardy and requires immediate attention from water management and planning.

## Declarations

### Author contribution statement

Bekan Chelkeba Tumsa, Msc, MA; Goshu Kenea Tujuba, Msc,; Bayisa Gedafa Kankure, Msc: Conceived and designed the experiments; Performed the experiments; Analyzed and interpreted the data; Contributed reagents, materials, analysis tools or data; Wrote the paper.

### Funding statement

This research did not receive any specific grant from funding agencies in the public, commercial, or not-for-profit sectors.

### Data availability statement

The data that has been used is confidential.

### Declaration of interest's statement

The authors declare no conflict of interest.

### Additional information

No additional information is available for this paper.
